# Sedentary behavior and depressive symptoms among 67,077 adolescents aged 12–15 years from 30 low- and middle-income countries

**DOI:** 10.1186/s12966-018-0708-y

**Published:** 2018-08-08

**Authors:** Davy Vancampfort, Brendon Stubbs, Joseph Firth, Tine Van Damme, Ai Koyanagi

**Affiliations:** 1 KU Leuven Department of Rehabilitation Sciences, Tervuursevest 101, 3001 Leuven, Belgium; 2 KU Leuven, University Psychiatric Center KU Leuven, Leuvensesteenweg 517, 3070 Kortenberg, Belgium; 3 Physiotherapy Department, South London and Maudsley NHS Foundation Trust, Denmark Hill, London, SE5 8AZ UK; 4 Health Service and Population Research Department, Institute of Psychiatry, Psychology and Neuroscience, King’s College London, De Crespigny Park, London, Box SE5 8AF UK; 5 NICM Health Research Institute, School of Science and Health, University of Western Sydney, Sydney, Australia; 6 Division of Psychology and Mental Health, Faculty of Biology, Medicine and Health, University of Manchester, Manchester, UK; 7 Centre for Youth Mental Health, University of Melbourne, Melbourne, Australia; 8 Research and Development Unit, Parc Sanitari Sant Joan de Déu, Universitat de Barcelona, Fundació Sant Joan de Déu, Dr. Antoni Pujadas, 42, Sant Boi de Llobregat, 0883 Barcelona, Spain; 9 Instituto de Salud Carlos III, Centro de Investigación Biomédica en Red de Salud Mental, CIBERSAM, Monforte de Lemos 3-5 Pabellón 11, 28029 Madrid, Spain

**Keywords:** Sedentary behavior, Sitting, Physical activity, Depression, Adolescents

## Abstract

**Background:**

Depression is common and burdensome in adolescents. Understanding modifiable environmental risk factors is essential. There is evidence that physical activity is protective of depression. However, the impact of sedentary behavior (SB) on depression is relatively under-researched especially in low- and middle-income countries (LMICs). In this cross-sectional study, we explored the association between SB and depressive symptoms in adolescents from 30 LMICs, controlling for confounders including physical activity.

**Method:**

Data from the Global school-based Student Health Survey were analyzed in 67,077 adolescents [mean (SD) age 13.8 (0.9) years; 50.6% girls). Self-report measures assessed depressive symptoms during the past 12 months, and SB, which was a composite variable assessing time spent sitting and watching television, playing computer games, talking with friends during a typical day excluding the hours spent sitting at school and doing homework. Multivariable logistic regression analysis was conducted and a countrywide meta-analysis undertaken.

**Results:**

The prevalence of depressive symptoms and ≥ 3 h/day of SB were 28.7 and 30.6%, respectively. There was a linear increase in the prevalence of depressive symptoms with increasing sedentary time beyond ≥3 h/day (vs. < 1 h/day). Among boys, 1–2 h/day of SB was associated with lower odds for depression (vs. < 1 h/day). Countrywide meta-analysis demonstrated that spending ≥3 h/day versus < 3 h/day was associated with a 20% increased odds for depressive symptoms (OR = 1.20; 95% CI = 1.16–1.24) with low between-country heterogeneity (*I*^*2*^ = 27.6%).

**Conclusion:**

Our data indicate that being sedentary for ≥3 h/day is associated with increased odds for depressive symptoms in adolescence. Future longitudinal data are required to confirm/refute the findings to inform public interventions which aim to limit the time spent being sedentary in adolescents.

## Background

Mental health disorders are a leading cause of disability worldwide [1], and onset is typically in adolescence [2]. Depression is the most common mental health disorder in adolescents [3]. The incidence, most notably in girls, rises quickly after puberty, and by the end of adolescence, the worldwide one-year prevalence rate is about 4% [4]. The burden is however the highest in low- and middle-income countries (LMICs) with prevalence rates in adolescents being reported to be as high as 28% [5]. Depression is a major risk factor for suicide in this age-group [6]. It also leads to serious social [7] and cognitive [8] impairments, school dropout [9], a higher risk for unhealthy lifestyle behaviors such as smoking [10] and alcohol abuse [11], and ultimately a higher risk of developing cardiovascular diseases early in life [12]. Therefore, from a public health perspective, it is essential to understand the risk factors that are associated with depressive symptoms in adolescents such that targeted interventions can be developed to assist in prevention and treatment.

Two related lifestyle behaviors that have been associated with depression, particularly in adults [13], are physical activity and sedentary behavior. While physical activity can be defined as any bodily movement produced by skeletal muscles that requires energy expenditure [14], sedentary behavior refers to any waking behavior characterized by an energy expenditure ≤1.5 metabolic equivalents, while in a sitting, reclining or lying posture [15]. Sedentary behavior is highly prevalent among adolescents. For example, a recent study [16] found that more than one third of 72,845 school-going adolescents from 34 different countries spend 3 or more hours per day in sedentary activities, excluding the hours spent sitting at school and doing homework, while only 23.8% of boys and 15.4% of girls meet the physical activity recommendations of at least 60 min of physical activity per day on at least 5 days per week.

The relationship between sedentary behavior, physical activity and depressive symptoms is complex. On one hand, depressive feelings can lead to disengagement in physical activity and increased engagement in sedentary behaviors [17], while on the other hand, recent evidence shows that avoiding sedentary behavior and engaging in physical activity can alleviate or prevent depressive symptoms in adolescents [18, 19].

In addition, a number of other gaps in the literature exist. First, few studies have examined associations between sedentary behavior and depressive symptoms among adolescents from LMICs [20]. Exploring associations between the presence of depressive symptoms and sedentary behavior in LMICs is important given different socio-cultural attitudes towards physical activity, different methods of transportation to and from school, and different environmental factors (e.g., safety, climate) in LMICs compared with high-income countries [21]. Second, most of the previous studies exploring associations between depression and sedentary behavior in adolescents did not adjust for physical activity levels [22]. This is an important omission, given the fact that recent large-scale data has demonstrated the protective effect of physical activity on depression [13]. Third, most previous studies on the sedentary behavior and depressive symptom relationship in adolescence are of small sample size, limited to a single country, and often restricted to a particular setting [22]. Furthermore, to date, multinational studies exploring these associations are absent. Multinational studies allow exploration of associations between sedentary behavior and depressive symptoms irrespective of national policies and available facilities, and at the same time allow comparison between countries in order to investigate the role of these policies and available facilities in different countries.

In order to address these gaps in the literature, we explored the association between sedentary behavior and depressive symptoms in 30 LMICs while taking into account physical activity levels. We hypothesize that in adolescents sedentary behavior is independently from physical activity participation associated with an increased odds for depressive symptoms.

## Methods

### The survey

Publically available data from the Global school-based Student Health Survey (GSHS) were analyzed. Details on this survey can be found at http://www.who.int/chp/gshs and http://www.cdc.gov/gshs. Briefly, the GSHS was jointly developed by the World Health Organization and the United States Centers for Disease Control and Prevention and other United Nations allies. The core aim of this survey was to assess and quantify risk and protective factors of major non-communicable diseases. The survey used a standardized two-stage probability sampling design for the selection process within each participating country. For the first stage, schools were selected with probability proportional to size sampling. The second stage involved the random selection of classrooms which included students aged 13–15 years within each selected school. All students in the selected classrooms were eligible to participate in the survey regardless of age. Data collection was performed during one regular class period. The questionnaire was translated into the local language in each country, and consisted of multiple choice response options. Students recorded their response on computer scannable sheets. All GSHS surveys were approved, in each country, by both a national government administration (most often the Ministry of Health or Education) and an institutional review board or ethics committee. Student privacy was protected through anonymous and voluntary participation, and informed consent was obtained as appropriate from the students, parents and/or school officials. Data were weighted for non-response and probability selection.

From all publically available data, we selected all datasets that included the variables pertaining to our analysis. The question on depressive symptoms was only available in the questionnaire for surveys administered between 2003 and 2008. If there were more than two datasets from the same country during this period, we chose the most recent dataset. A total of 30 countries were included in the current study. Based on the World Bank classification at the time of the survey, all countries were LMICs. Data were nationally representative for all countries with the exception of six countries where the survey was only conducted in selected areas: Chile (Metropolitan areas), China (Beijing), Ecuador (Quito), Tanzania (Dar Es Salaam), Venezuela (Lara), and Zimbabwe (Harare). The characteristics of each country including the sample size and the response rate are provided in Table 1.

**Table 1:** Sample characteristics by country

Country	N	Year	Response rate (%)	Depressive symptoms (%)	≥3 h/day sedentary (%)
Argentina	1537	2007	77.1	29.5	48.7
Botswana	1397	2005	95.0	39.0	34.6
Chile (Metropolitan)	1972	2004	85.0	30.8	44.4
China (Beijing)	2189	2003	99.0	18.3	22.0
Djibouti	962	2007	83.3	37.5	32.3
Ecuador (Quito)	1842	2007	85.6	25.1	28.7
Egypt	4981	2006	87.0	35.1	23.1
Grenada	1299	2008	78.0	23.4	41.1
Guyana	1070	2004	80.0	32.5	36.3
India	7330	2007	84.2	24.4	22.8
Indonesia	3022	2007	93.1	21.0	33.8
Jordan	1648	2007	99.8	37.1	38.2
Kenya	2971	2003	83.5	47.8	37.7
Montserrat	161	2008	78.0	26.7	47.0
Morocco	1986	2006	84.0	36.5	29.9
Myanmar	2227	2007	95.0	14.7	9.7
Namibia	4529	2004	81.7	38.3	30.7
Philippines	3484	2007	81.0	36.6	29.5
St. Lucia	1072	2007	82.0	23.3	52.6
St. Vincent & the Grenadines	1188	2007	84.0	27.5	39.1
Seychelles	1154	2007	82.0	32.0	51.4
Sri Lanka	2504	2008	89.0	31.8	33.2
Tanzania (Dar Es Salaam)	1757	2006	87.0	23.7	29.0
Thailand	2675	2008	93.0	16.6	37.6
Tunisia	2549	2008	83.0	35.9	23.9
Uganda	1904	2003	68.4	39.3	27.4
Uruguay	2882	2006	71.2	16.6	49.6
Venezuela (Lara)	1970	2003	85.4	22.2	27.7
Zambia	1365	2004	70.5	51.5	32.6
Zimbabwe (Harare)	1450	2003	84.0	36.0	43.7

### Depressive symptoms (outcome)

Those who answered affirmatively to the question “During the past 12 months, did you ever feel so sad or hopeless almost every day for two weeks or more in a row that you stopped doing your usual activities?” were considered to have depressive symptoms [21].

### Sedentary behavior (exposure)

Sedentary behavior was assessed with the question “How much time do you spend during a typical or usual day sitting and watching television, playing computer games, talking with friends, or doing other sitting activities?” with answer options: < 1, 1–2, 3–4, 5–6, 7–8, and ≥ 8 h/day. This excluded time at school and when doing homework. This variable was used as a six-category variable or a dichotomized variable (≥3 h/day or not), in accordance with previous research [16].

### Control variables

Sex, age, and physical activity were used as control variables in the analysis. To assess levels of physical activity, questions that represented the PACE+ Adolescent Physical Activity Measure [23] were asked. This measure has been tested for validity and reliability [23]. The questions asked about the number of days with physical activity of at least 60 min during the past 7 days and during a typical week. This did not include physical activity during physical education or gym classes. The number of active days during the past week and a typical week were averaged. We also considered socioeconomic status as a potential confounder. Although there are no variables on socioeconomic status in the GSHS, previous GSHS studies have used food insecurity as a proxy measure [24]. Specifically, this was assessed by the question “During the past 30 days, how often did you go hungry because there was not enough food in your home?” However, preliminary analysis showed that results remain largely unchanged even after adjustment for this variable. Thus, we decided to omit this variable from the analysis.

### Statistical analysis

Statistical analyses were performed with Stata 14.1 (Stata Corp LP, College station, Texas). The analysis was restricted to those aged 12–15 years as most students were within this age range and data on the exact age out of this age range was not provided. Multivariable logistic regression analysis was conducted to assess the association between time spent in sedentary behavior (exposure) and depressive symptoms (outcome). The exposure variable was the six-category sedentary behavior variable when the overall or sex-stratified samples were used. However, for country-wise analyses, we used the dichotomized sedentary behavior variable to obtain stable estimates, as the sample size in each country was small. The regression analyses were adjusted for age, sex, physical activity, and country with the exception of the sex-stratified and country-wise analyses which were not adjusted for sex and country, respectively. Adjustment for country was done by including dummy variables for each country [25, 26]. To assess the level of between-country heterogeneity, the Higgin’s *I*^*2*^ statistic was calculated based on country-wise estimates. This represents the degree of heterogeneity that is not explained by sampling error with a value of < 40% often considered as negligible and 40–60% as moderate heterogeneity [27]. A pooled estimate was obtained by combining the estimates for each country into a fixed effect meta-analysis.

All variables were included in the regression analysis as categorical variables with the exception of age and physical activity (continuous variable). Under 3% of the data were missing for all the variables used in the analysis. Complete case analysis was done. Taylor linearization methods were used in all analyses to account for the sample weighting and complex study design. Results from the logistic regression analyses are presented as odds ratios (ORs) with 95% confidence intervals (CIs). The level of statistical significance was set at *p* < 0.05.

## Results

**Fig. 1:**
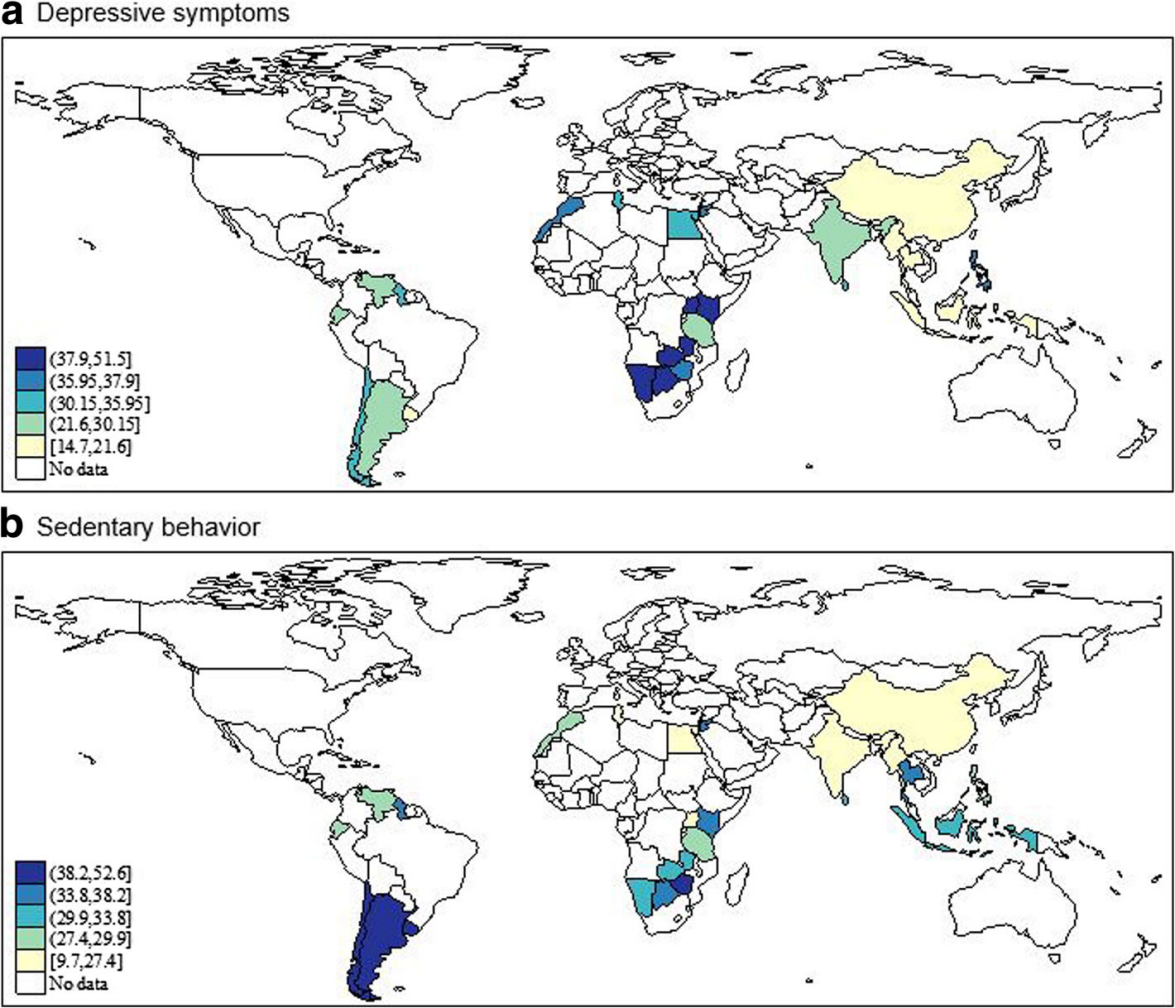
Prevalence of (**a**) depressive symptoms and (**b**) ≥3 h/day of sedentary behavior by country Data from China, Chile, Ecuador, Tanzania, Venezuela, and Zimbabwe were from selected sites

The final sample consisted of 67,077 adolescents aged 12–15 years with a mean (SD) age of 13.8 (0.9) years and 50.6% were females. The overall prevalence of depressive symptoms was 28.7%. Sedentary behavior of ≥3 h/day was observed in 30.6% of the sample (ranging from 9.7% in Myanmar, to 52.6% in St. Lucia). The prevalence of sedentary behavior of < 1, 1–2, 3–4, 5–6, 7–8, and > 8 h/day were 32.9, 36.5, 18.2, 6.5, 2.2, and 3.7%, respectively. Many countries in Sub-Saharan Africa had a high prevalence of depressive symptoms, while sedentary behavior was highly prevalent in countries of the Americas (Fig. 1, Table 1).

**Fig. 2:**
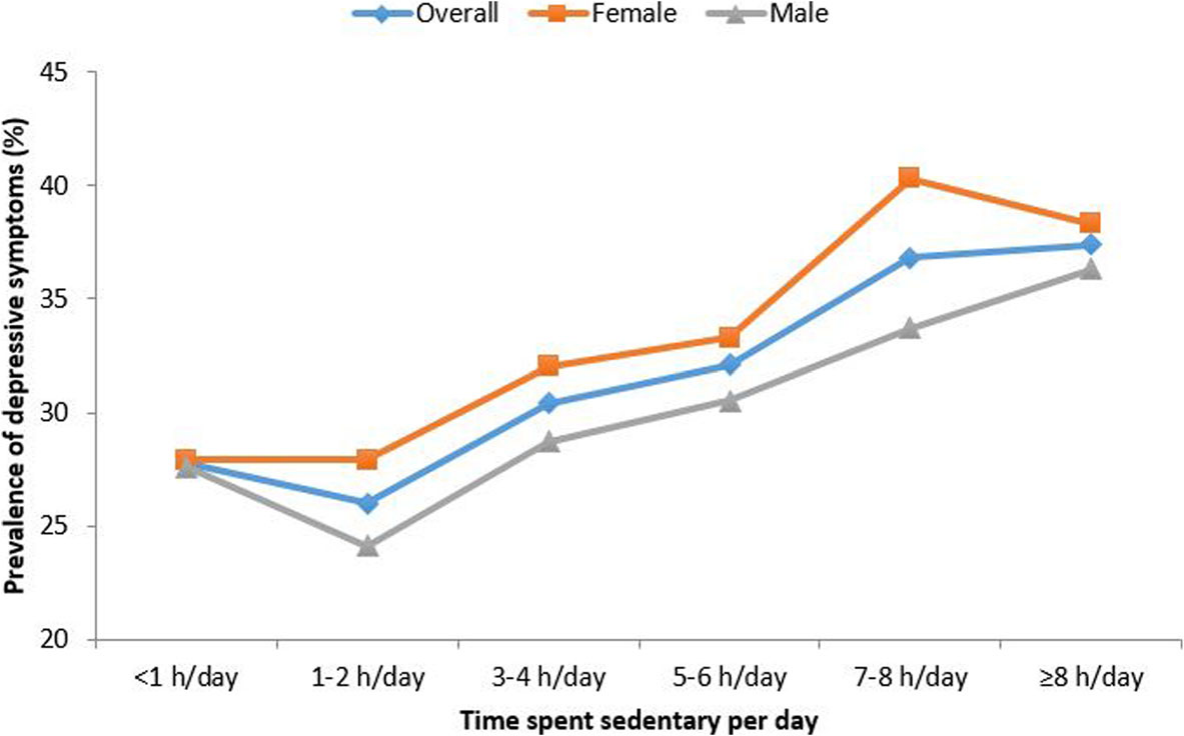
Prevalence of depressive symptoms by hours spent sedentary per day

Overall, there was a slight drop in the prevalence of depressive symptoms for those engaging in 1–2 h of sedentary behavior per day compared to < 1 h/day. This decrease was particularly pronounced among males (Fig. 2). Beyond 1–2 h/day, there was a linear increase in the prevalence of depressive symptoms with increasing sedentary time.

**Fig. 3:**
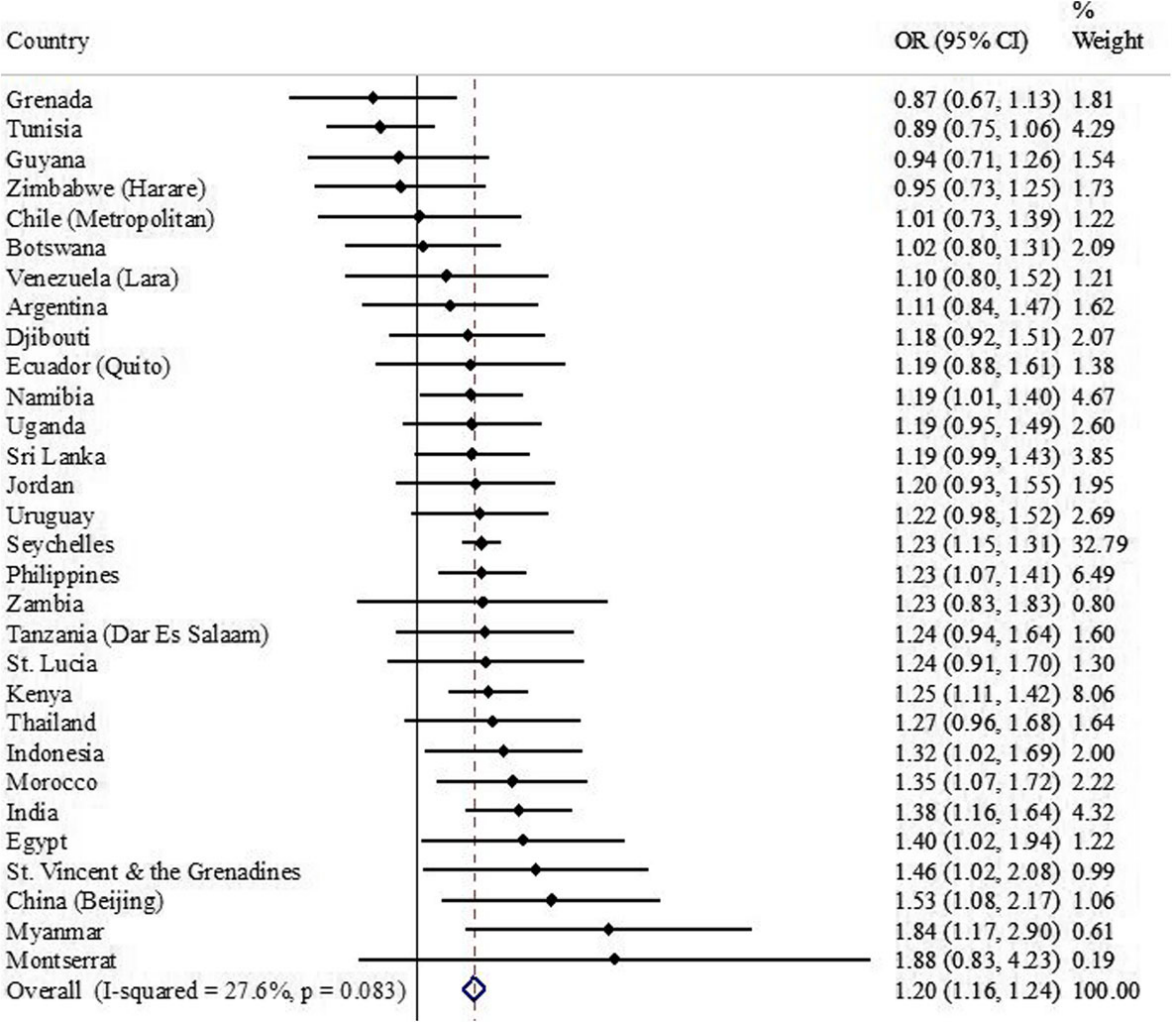
Association between ≥3 h of sedentary behavior per day (exposure) and depressive symptoms (outcome) estimated by multivariable logistic regression. Abbreviations: *OR* Odds ratio, *CI* Confidence interval. Models are adjusted for age, sex, and physical activity. The pooled estimate was calculated by meta-analysis with fixed effects

These findings were confirmed in adjusted analyses controlling for potential confounders including physical activity (Table 2). Adjusted analyses in the overall sample showed that compared to < 1 h/day of sedentary behavior, ≥3 h were associated with significant increasing odds for depressive symptoms. Similar trends were observed for females, while for males, compared to < 1 h/day of sedentary behavior, 1–2 h/day was associated with a significant 14% decreased odds for depressive symptoms (OR = 0.86; 95% CI = 0.77–0.97), and > 8 h/day, a 1.48 times higher odds (OR = 1.48; 95% CI = 1.19–1.85) (Table 2). Country-wise analysis showed that sedentary behavior of ≥3 h (vs. < 3 h/day) was associated with depressive symptoms (i.e., OR > 1) in 26 countries with statistical significance being reached in 11 countries (Fig. 3). Only a low level of between-country heterogeneity was observed (*I*^*2*^ = 27.6%) with the overall estimate based on a meta-analysis being 1.20 (95% CI = 1.16–1.24).

**Table 2:** Association between sedentary time and depressive symptoms estimated by multivariable logistic regression (overall and by sex)

	Overall^a^		Female^b^		Male^b^	
Time spent sedentary	OR	95% CI	OR	95% CI	OR	95% CI
< 1 h/day	1.00		1.00		1.00	
1–2 h/day	0.94	[0.86,1.02]	1.01	[0.91,1.13]	0.86*	[0.77,0.97]
3–4 h/day	1.15**	[1.05,1.27]	1.19**	[1.05,1.36]	1.09	[0.96,1.24]
5–6 h/day	1.25**	[1.09,1.44]	1.30**	[1.08,1.57]	1.19	[0.96,1.47]
7–8 h/day	1.44*	[1.07,1.92]	1.63**	[1.19,2.24]	1.25	[0.86,1.81]
> 8 h/day	1.53***	[1.30,1.81]	1.57***	[1.26,1.95]	1.48***	[1.19,1.85]

Abbreviation: OR Odds ratio; CI Confidence interval

^a^Adjusted for age, sex, physical activity, and country

^b^Adjusted for age, physical activity, and country

*p < 0.05, ** *p* < 0.01, *** *p* < 0.001

## Discussion

### General findings

The most consistent finding of the present study was that beyond 1–2 h/day, in both boys and girls, there was a linear increase in the prevalence of depressive symptoms with increasing sedentary time and this was irrespective of physical activity levels. Among boys, 1–2 h/day of sedentary behavior was significantly associated with a lower odd of depressive symptoms compared to < 1 h/day. Country-wise analysis showed that sedentary behavior of ≥3 h/day (vs. < 3 h/day) was associated with depressive symptoms (i.e., OR > 1) in 26 of the 30 included countries and only a low level of between-country heterogeneity was observed with the overall estimate based on a meta-analysis being 1.20 (95% CI = 1.16–1.24). Our study from LMICs supports previous findings from high-income single countries [28, 29] showing a linear association between sedentary behavior and worse mental health in adolescents, at least beyond 1–2 h/day of sedentary behavior.

Of interest and also in agreement with data from only high-income countries, is the slight drop in the prevalence of depressive symptoms for boys engaging in 1–2 h of sedentary behavior per day compared to < 1 h/day. One hypothesis is that more than one hour spent sedentary, for example viewing TV, might be a measure-of-proxy for a higher socioeconomic status (having a TV at home) in LMICs. Those without access to a TV might be less sedentary, but might also have a lower socioeconomic status, which is a known risk factor for depression in adolescents [30]. Another explanation could be related to screen behaviors enhancing adolescents’ ability to read and visualize images and, consequently, improving their academic performance. Improved academic performance might increase adolescents’ self-esteem [31] and a higher self-esteem is associated with a lower risk for developing depressive symptoms [32]. Alternatively, adolescents may benefit psychologically from processing humorous content when watching some, but not a lot of TV (i.e., for example 1–2 h/day) or playing computer games [22]. A difference with previous findings from high-income countries is that this lower depression risk for those engaging in 1–2 h of sedentary behavior per day compared to < 1 h/day was only observed in boys. The reason why this higher risk was found only in boys remains to be explored. It might be hypothesized that girls from poor families are less likely to attend school than boys as they are more prone to child labor in order to contribute to the family income and therefore might be proportionally under-represented in the GSHS. However, gender differences for associations between child labor and school enrollment seems to differ largely between countries and between different types of child labor [33].

Due to the cross-sectional nature of our study, clearly future longitudinal research is required to confirm the directionality of the relationships we observed and elucidate if a potential causal relationship may exist between sedentary behavior and depressive symptoms. However, previous research from randomized controlled trials in Western samples has suggested a causal relationship demonstrating the independent deleterious impact of increasing sedentary behavior on mood and in particular symptoms of anxiety in active youth [34], possibly through changes in inflammation [35], which is a core feature of major depression [36]. Previous research has also suggested that sedentary behavior is, for example, associated with higher c-reactive protein and interleukin 6 levels [37]. There is some provisional evidence in adults to suggest that standing and breaking up prolonged periods of sedentary behavior can improve inflammatory biomarkers profiles [38], while in adolescents metabolic benefits were observed [39]. In addition, adolescents may be more vulnerable to physiological responses from arousal of the central nervous system and associated negative effects on sleep patterns with high rates of screen viewing [40].

### Limitations, strengths and future research

The current findings should be interpreted in light of some limitations. First, and as stated, the study is cross-sectional. Therefore, the directionality of the relationships cannot be deduced with certainty. Longitudinal studies are required to better disentangle the relationships observed. Second, we used the GSHS data, which surveys adolescents in schools. Thus, the results might not necessarily reflect physical activity and sedentary behavior patterns among all adolescents. Specifically, information from adolescents who are unable to attend school or have dropped out, were not captured in this study but should be a target in future research endeavors. Third, the variable assessing depressive symptoms was based on a single self-report question for which the sensitivity and specificity against the gold standard diagnostic criteria is unclear. Fourth, physical activity and sedentary behavior were also only captured with a self-report measure, the accuracy of which has been questioned in pediatric populations [41]. Additionally, inclusion of information on school physical education and gym would have more comprehensively assessed physical activity in adolescents. Besides this, time spent sedentary excluded time at school and when doing homework and therefore is an underestimate of the real time spent sedentary during the entire day. Future research should utilize objective measures of sedentary behavior. Accelerometers-inclinometers are available that allow for valid and reliable assessment of sedentary behavior. However, the association between sedentary behavior and depressive symptoms may be dependent on the domain/type of sedentary behavior (e.g., cognitively active sedentary behavior, such as reading and internet use, versus cognitively passive TV viewing) [42], an aspect that is not reliably measured with accelerometers. Therefore, a combination of both objective and subjective methods is warranted.

Nonetheless, the strengths of the study include the largest sample size to date on this topic (over 67,000) and the multi-national scope. Most of the research in the domain of sedentary behavior and depressive symptoms has been conducted in Western countries, and little is known about it in regions across where there are multiple economic, cultural or social factors or differences in the health systems. The present study was furthermore performed with mostly nationally representative samples of adolescents attending school.

Given the findings of our study, but also the wider literature from longitudinal research considering the deleterious impact of sedentary behavior on multiple health outcomes in adolescents [43], it is essential that public health interventions should seek to limit the time spent sedentary in this young population.

## Conclusions

Our paper provides multi-national evidence of a relationship between sedentary behavior and depressive symptoms in adolescents and this was irrespective of physical activity levels. The consistency of these relationships observed, at least beyond 1–2 h/day of sedentary behavior, across the included countries adds further weight to the growing evidence for a connection between sedentary behavior and poor mental health in adolescents. Following future longitudinal studies, these findings could offer important new targets and strategies for interventions to tackle the depression-sedentary behavior relationship at its very early stages.

## Abbreviations

CIs: confidence intervals
GSHS: Global school-based Student Health Survey
LMICs: low- and middle-income countries
ORs: odds rations
